# Multiplex Networks to Characterize Seizure Development in Traumatic Brain Injury Patients

**DOI:** 10.3389/fnins.2020.591662

**Published:** 2020-11-30

**Authors:** Marianna La Rocca, Rachael Garner, Nicola Amoroso, Evan S. Lutkenhoff, Martin M. Monti, Paul Vespa, Arthur W. Toga, Dominique Duncan

**Affiliations:** ^1^Laboratory of Neuro Imaging, USC Stevens Neuroimaging and Informatics Institute, Keck School of Medicine, University of Southern California, Los Angeles, CA, United States; ^2^Dipartimento di Farmacia - Scienze del Farmaco, Università degli Studi di Bari “A. Moro”, Bari, Italy; ^3^Department of Psychology, University of California, Los Angeles, Los Angeles, CA, United States; ^4^David Geffen School of Medicine, University of California, Los Angeles, Los Angeles, CA, United States

**Keywords:** post-traumatic epilepsy, traumatic brain injury, structural magnetic resonance imaging, multiplex networks, random forest, machine learning, complex networks

## Abstract

Traumatic brain injury (TBI) may cause secondary debilitating problems, such as post-traumatic epilepsy (PTE), which occurs with unprovoked recurrent seizures, months or even years after TBI. Currently, the Epilepsy Bioinformatics Study for Antiepileptogenic Therapy (EpiBioS4Rx) has been enrolling moderate-severe TBI patients with the goal to identify biomarkers of epileptogenesis that may help to prevent seizure occurrence and better understand the mechanism underlying PTE. In this work, we used a novel complex network approach based on segmenting T1-weighted Magnetic Resonance Imaging (MRI) scans in patches of the same dimension (network nodes) and measured pairwise patch similarities using Pearson's correlation (network connections). This network model allowed us to obtain a series of single and multiplex network metrics to comprehensively analyze the different interactions between brain components and capture structural MRI alterations related to seizure development. We used these complex network features to train a Random Forest (RF) classifier and predict, with an accuracy of 70 and a 95% confidence interval of [67, 73%], which subjects from EpiBioS4Rx have had at least one seizure after a TBI. This complex network approach also allowed the identification of the most informative scales and brain areas for the discrimination between the two clinical groups: seizure-free and seizure-affected subjects, demonstrating to be a promising pilot study which, in the future, may serve to identify and validate biomarkers of PTE.

## 1. Introduction

Traumatic brain injury (TBI) is the third most common cause of death and debilitating secondary problems in adults and children worldwide. One common consequence of TBI that causes significant disability amongst patient populations is post-traumatic epilepsy (PTE) (Humphreys et al., 2013). This condition develops in up to 50% of patients with TBI. Post-traumatic epilepsy (PTE) is diagnosed if two or more unprovoked seizures occur at least 1 week after a TBI (Diaz-Arrastia et al., 2009). Recent investigations suggest that injury severity and especially epileptic activity are high risk factors of PTE, although the mechanisms by which trauma to the brain tissue leads to recurrent seizures is not known. Therefore, studying if specific structural Magnetic Resonance Imaging (sMRI) changes can be related to seizures after a TBI is of fundamental importance to carry out the first steps toward the discovery of early biomarkers of PTE (Kim et al., 2018). PTE is not a homogeneous condition and can appear weeks or several years after a TBI. As a consequence, the precise percentage of TBI patients who develop PTE is not known (Verellen and Cavazos, 2010). Currently, growing attention has been devoted to investigate PTE. In this regard, the Epilepsy Bioinformatics Study for Antiepileptogenic Therapy (EpiBioS4Rx) is an international, multi-center project conceived to identify biomarkers of epileptogenesis after a TBI in order to evaluate treatments that could prevent the development of PTE and design clinical trials of antiepileptogenic therapies on an extensive patient population. With this project, the scientific community can be granted access to a large amount of high quality, multi-modal data, including imaging, electrophysiology, and clinical data from both humans and animals.

Changes in gray matter and white matter related to epilepsy have been widely observed by using structural MRI (Immonen et al., 2018; Shah et al., 2019; Lutkenhoff et al., 2020). Many recent studies have shown that machine learning techniques and multiplex networks applied to completely non-invasive neuroimaging techniques, such as structural MRI, can be useful and efficient to detect pathological alterations in several neurological diseases, such as Alzheimer's disease, Parkinson's disease, and epilepsy (Amoroso et al., 2018c; La Rocca et al., 2018; Bharath et al., 2019). Multiplex networks overcome the limit of the existing complex network standard approaches not to be able to collectively study what happens to the same nodes as their interactions change. In our previous work (Garner et al., 2019), we used different machine learning strategies to identify alterations in functional brain connectivity that are related to seizure outcome following TBI. However, the present study is the first which uses the combination of multiplex networks of structural MRIs and machine learning techniques to distinguish patients who have developed at least one seizure after a TBI from those who have not experienced any seizures. This study is of paramount importance, because it offers an opportunity to observe alterations in TBI brain networks that may reflect structural MRI changes related to seizure development.

This paper provides three main results: (i) the implementation of a pipeline which combines complex network and machine learning models for the identification of TBI patients who have developed epilepsy; (ii) the investigation of the most appropriate scale or patch size to study seizure development in TBI patients; (iii) the implementation, on a TBI cohort, of a promising complex network model based on segmenting the brain in patches to obtain comprehensive clinical information on the whole brain. In the future, this pilot study may help clinicians localize the epileptogenic focus more precisely, relate brain lesions to seizure occurrence and understand the relationship between neuronal activity abnormalities and structural damage.

## 2. Materials and Methods

### 2.1. Dataset

In this work, we used 53 structural MRI scans of TBI subjects recruited in EpiBioS4Rx according to specific inclusion and exclusion available online^1^. Sixteen of these subjects have experienced at least one seizure within 6 months of a TBI and 37 have not experienced any seizures. As part of their clinical care, 14 subjects required a craniectomy (3 seizure and 11 non-seizure, *p* > 0.05). Additional clinical and demographic information are reported in Tables 1, 2. 3D T1-weighted volumes were acquired within 32 days (median 8 and interquartile range of [2, 15]) after the TBI using 3T Siemens, Philips, and GE scanners according to a magnetization-prepared rapid acquisition gradient echo (MPRAGE) sequence with the following parameters: 256 mm field of view (FOV); 1 mm slice thickness; 1,500–2,500 ms repetition time (TR); minimum echo time (TE); 1,100–1,500 ms inversion time (TI); 8–15 degree flip angle; 256 phase-encoding steps, number of excitations (NEX) >1 and 256 Hz frequency.

**Table 1:** Imaging findings are reported for each clinical class.

**Injury type**	**Seizure-free patients**	**Patients with seizure**
Skull fracture	27/37	16/16
Epidural hematoma	8/37	4/16
Extraaxial hematoma	18/37	9/16
Acute subdural hematoma	27/37	14/16
Subarachnoid hemorrhage	30/37	14/16
Intracerebral/Intraparenchymal hemorrhage	22/37	11/16
Midline shift (Avg shift)	21/37(4.53)	8/16(6.47)
Cisternal compression	6/37	3/16
Frontal contusion	22/37	9/16
Temporal contusion	19/37	7/16
Brain edema	14/37	7/16
Penetrating injury	1/37	0/16

*Injury characteristics were reported by clinical staff based on patient Computed Tomography (CT) scans on the day of hospital admission. No statistically significant between-group differences were found in the imaging findings except in the skull fracture (p = 0.02)*.

**Table 2:** Sample size, gender, and Glasgow Coma Score (GCS) information are reported for each clinical class.

**Clinical status**	**Sample size**	**Age**	**Female/Male**	**GCS score**
Seizure-free patients	37	36.28 ± 21.18	4/33	10.78 ± 4.05
Patients with seizure	16	40.50 ± 18.05	3/13	8.94 ± 3.59

*Age and GCS were provided in terms of mean and standard deviation. No statistically significant differences between the two classes were found with respect to age, GCS score, and gender. Statistical evaluations were performed with a Kruskal–Wallis statistic test except for the gender, for which a Chi-square test was used*.

### 2.2. MRI Processing

**Figure 1:**
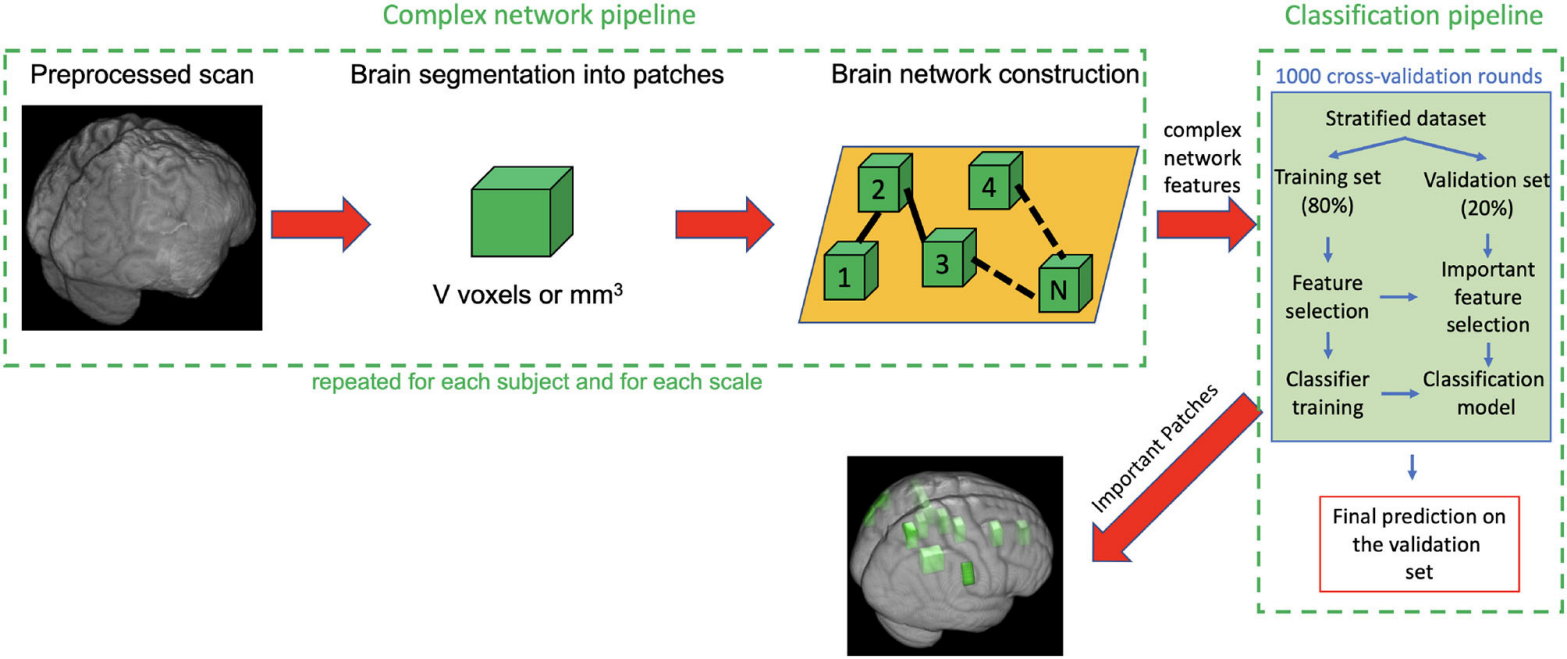
Description of the complex network pipeline and machine learning pipeline. First each subject scan is preprocessed, segmented into patches, then for each subject a weighted undirected network was built and some complex network features were computed. Finally, the feature representation (subject × network features), obtained after the removal of null mean and variance features and highly correlated features, was used as input for the classification pipeline. The machine learning pipeline includes 1,000 rounds of cross-validation (CV). In each round the following steps are performed: (i) dataset was stratified; (ii) 80% of the stratified dataset was used as training set and 20% as validation set; (iii) training set was used, through a first nested Random Forest (RF) classifier, to select the most important features for the discrimination of seizure-free and seizure affected subjects: (iv) these selected features were used in turn to train a second RF classifier; (v) the important features and the classification models obtained on the training test were used to classify the subjects of the validation set; (vi) Averaging the classification performances over the 1,000 CV rounds, we obtained the final accuracy sensitivity, specificity, area under the receiver operating characteristics curve (AUC) and confidence interval on the validation test.

Protocol compliance and quality control (QC) were undertaken using the Laboratory Of Neuro Imaging (LONI) QC System^2^. Structural MRI scans were processed with the Oxford FMRIB Software Library (FSL) (Jenkinson et al., 2012). Firstly, image skull-stripping was obtained with the optimized brain extraction script for patient brain (optiBET) (Lutkenhoff et al., 2014). For those few cases where the automatic brain extraction was particularly challenging due to the significant brain deformation caused by the trauma, we performed the skull stripping by manually adjusting the brain extraction threshold with FSL Brain Extraction Tool (BET). Then MRI scan intensity differences, yielded by bias field, were normalized. After intensity normalization and brain extraction, a spatial normalization was performed to co-register the different images into a common coordinate space by using an affine transformation. The MNI152 was adopted as the reference template, and registration was performed with the FSL Linear Registration Tool (FLIRT) with a standard parameter configuration. Even though a deformable registration would have given a better overlap among TBI subjects, we purposely used an affine registration for three main reasons: (i) proving the robustness of the method also in case of roughly overlap between the anatomical regions of different TBI subjects; (ii) avoiding misregistration issues due to the particularly challenging process to apply a non linear transformation to a cohort with huge brain deformations; (iii) registering all the subjects to a common reference space keeping as much as possible the individual differences of the subjects and the relative lesions. Besides these initial steps, the analysis pipeline includes two principal sub-pipelines: a complex network pipeline and a machine learning pipeline that are schematized in Figure 1 and are described in detail in the next two sections.

### 2.3. Multiplex Network Pipeline

**Figure 2:**
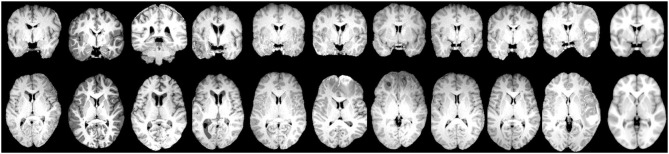
Examples of coronal plane **(Top)** and axial plane **(Bottom)** of TBI subjects with the most severe imaging findings **(Left)** and the MNI152 reference template **(Right)** which the subjects' images are aligned to.

After image processing, each scan was parceled in homologous non-overlapping parallelepipeds or patches of V voxels (where 1 voxel is 1 cubic millimeter) in order to obtain a 3D grid. These patches represent nodes of a brain network, and the absolute values of Pearson's correlation between patch pairs were considered the links between the nodes. In other words, each network link is obtained by computing the correlation voxel-by-voxel between the T1 intensities of two patches. Therefore, for each scan, we obtained a weighted brain network using a patch-based segmentation. To remove links due to the noise, we neglected all the correlations lower than 0.3. This threshold has not been chosen arbitrarily but has been demonstrated in our previous works (Amoroso et al., 2018b,c) as a threshold that maximizes classification performance and is the best trade-off between minimizing noise and maintaining effective network information in this multiplex network methodology. This threshold choice is also confirmed by other works in literature, for example, Mukaka (2012) suggests that correlations lower than 0.3 are negligible in his guide about the appropriate use of correlation coefficients in medical research. To further avoid false positive links in the networks, we also excluded the patches with a non-brain number of voxels exceeding 10% of their volume. The idea behind this study is that seizure development in TBI patients may be related to injury severity which, as many study demonstrate, results in diffuse cerebral edemas, hemorrhages, contusions, and distortions of brain tissue localized in multiple brain regions both close to and distant from the lesion area. Patch-based approach is aimed to detect this alterations in terms of correlation variation between regions with and without tissue damage over the TBI cohort (Kurland et al., 2012). A patch-based approach is a beneficial trade-off between a voxel-based approach and an ROI-based approach and has already been found to be beneficial in the field of other neurodegenerative diseases (Suk et al., 2014). It has three main advantages: (i) it overcomes the problem of the “curse of dimensionality,” (ii) it does not depend on segmentation accuracy, and (iii) it is robust to misregistration errors (Amoroso et al., 2018a). Therefore, the patch representation can be very useful for TBI patients for whom ROI segmentation and spatial registration are particularly challenging tasks due to the large and irregular brain deformations caused by TBI lesions. To give a sense of the injury severity and the related processing challenges that were faced for this cohort, in Figure 2, for some of the TBI subjects with the most severe imaging findings, axial and coronal planes of brain scans after the processing (brain extraction and registration) are represented alongside the template that the subjects' scans are registered to.

The size V of each patch was varied from 1,000 to 8,000 voxels in steps of 1,000 to investigate the most appropriate scales to study seizure development in TBI patients. At different scales, the patch number is not constant but is determined by the patch size. The grid's origin is fixed for all the scales because we start to segment each image from the medial sagittal plane which separates the two brain hemispheres in order to uniformly cover each hemisphere with an equal number of rectangular boxes. For each scale, we built a multiplex network *G* = {*G*_1_, *G*_2_, ..., *G*_*i*_, ..., *G*_*M*_} that is a collection of single subjects' weighted networks *G*_*i*_ = (*N, E, W*) sharing the same nodes N, while the set of links E and weights W change depending on the subject's brain networks connections or layer connections. In other words, in each multiplex network, the same number of nodes (patches that each scan is segmented into) can be connected in different ways depending on the specific correlation coefficient values that characterize the network connections of a certain layer. Then, given N, the number of network nodes, we obtained 8*N* features for each subject, 4*N* features of single layer and 4*N* features of multiplex networks. The single layer features used in this work are strength and inverse participation ratio, given by the Equations (1) and (2), and their conditional means over the nodes with the same degree k, thus having the same connection number. Conditional means, given by the Equations (4) and (5), can be useful to examine whether, on average, the weights of central nodes and less connected nodes are identically distributed.

(1) $$\begin{array}{r} s_{i}^{\alpha }=\overset{N}{\underset{j=1}{\sum }}w_{ij}^{\alpha } \end{array}$$

(2) $$\begin{array}{r} y_{i}^{\alpha }=\overset{N}{\underset{j=1}{\sum }}{\left(\frac{w_{ij}^{\alpha }}{s_{i}^{\alpha }}\right)}^{2} \end{array}$$

(3) $$\begin{array}{r} s{\left(k\right)}^{\alpha }=\frac{1}{N_{k}}\overset{N}{\underset{i=1}{\sum }}s_{i}^{\alpha }\delta \left(k_{i}^{\alpha },k\right) \end{array}$$

(4) $$\begin{array}{r} Y{\left(k\right)}^{\alpha }=\frac{1}{N_{k}}\overset{N}{\underset{i=1}{\sum }}Y_{i}^{\alpha }\delta \left(k_{i}^{\alpha },k\right) \end{array}$$

α = 1, .., *M* indicates the network layer, *w*_*i*_*j* is the correlation between gray level intensities of the nodes *i, j* = 1, .., *N* with M subject number, *N* is the node number, *N*_*k*_ is the number of nodes having degree *k*, and δ is the Kronecker Delta function. Strength and inverse participation ratio indicate, respectively, the importance of a node and how evenly distributed the connections between nodes are. Specifically, ${\left(y_{i}^{\alpha }\right)}^{-1}\in \left(1,k_{i}^{\alpha }\right)$ has value $k_{i}^{\alpha }$ if the weights of the links of node *i* are distributed uniformly and, it has value 1 if the weight of one link is much larger than the other weights (Bianconi, 2018). The multiplex network features were obtained by weighing the previous quantities on the multiplex network degree *k*_*multi*_, given by (5), indicating the number of connections of a node in the multiplex network.

(5) $$\begin{array}{r} k_{i}^{multi}=\overset{N}{\underset{j=1}{\sum }}a_{ij}^{multi}; \end{array}$$

where $a_{ij}^{multi}$ is 1 if there is a layer with at least one link between nodes i and j and zero otherwise as described in Amoroso et al. (2018b). From now on, we will refer to these multiplex quantities as multi-strength, multi-inverse participation ratio, conditional strength, and conditional multi-inverse participation ratio. Overall, we obtained for each scale V, a M (subject network number) X 8N feature representation to analyze with the machine learning pipeline described in the next section.

### 2.4. Machine Learning Pipeline

For each scale V, multiplex network features were used to train a Random Forest (RF) classifier and obtain reliable classification models to identify, on the validation set, which TBI patients have developed seizures and which have not. This classification process, preceded by the removal of null mean and variance features, and highly correlated features (>0.95), was carried out within a machine learning pipeline that includes 1,000 rounds of stratified cross-validation (Vabalas et al., 2019). For each round, we randomly picked the same percentage of seizure-free subjects and seizure-affected subjects in order to examine balanced datasets. After the stratification, a training set (80% of the stratified set) and validation set (20% of the stratified set) were defined. Subsequently, we first used a nested RF classifier, on the training set, to select and record features exceeding the third quartile of the importance distribution computed in terms of mean accuracy decrease. Then, we used those features to train a second RF classifier and obtain the classification models. Feature selection and training phases were nested within each cross-validation round and were blind to the validation set to avoid the “double dipping” problem (Kriegeskorte et al., 2009). Finally, we used the classification models and the important features retrieved during the training phase to classify the two clinical classes. Classification performances for each scale were evaluated in terms of accuracy, specificity, sensitivity, and Area Under the receiver-operating-characteristic Curve (AUC) averaged over all the cross-validation rounds. For the average accuracy, we also reported the 95% confidence interval computed according to the Wilson score interval (Wilson, 1927). We chose to use RF model because it is a robust and easy-to-tune model, it does not overfit thanks to internal bagging and it is particularly appropriate for analyses with high-dimensional feature spaces and small sample sizes (even <100) (Biau and Scornet, 2016; Floares et al., 2017). Each forest was grown with 500 trees, a number large enough for the out-of-bag error to reach the typical training plateau (Breiman, 1996). Therefore, in the internal bagging, given the training set, 500 bootstraps are formed obtaining 500 new subjects sets used to grow 500 trees. Each tree is grown by randomly choosing a subset of features equal to the square root of the feature number. The learning model built in this way can be then used to compute the out-of-bag error and the accuracy on the data left out of the training set.

### 2.5. Important Feature Assessment

After having found for each round the important features, we evaluated, for the best scales, which feature occurrences had not happened by chance over the 1,000 rounds by using the statistical test of equal or given proportions (Newcombe, 1998). Therefore, the most important features over all the cross-validation rounds were found by considering, after the Bonferroni correction for multiple comparison, a *p*−*value* < α*N^−1^ with α = 0.05. From the most important nodal features, it was possible to find the most important network nodes or patches, and thus the most important anatomical regions. We considered an anatomical region significantly related to the seizure development only if it occupied an important patch with a volume greater than 10% of the patch voxels. To identify the most important anatomical regions, we used Talairach labels projected in MNI 152 space (Lancaster et al., 2000). Other details on the reliability of the feature selection methods used in this work are discussed in the Supplementary Material.

## 3. Results

### 3.1. Classification Performance and Feature Evaluation

**Figure 3:**
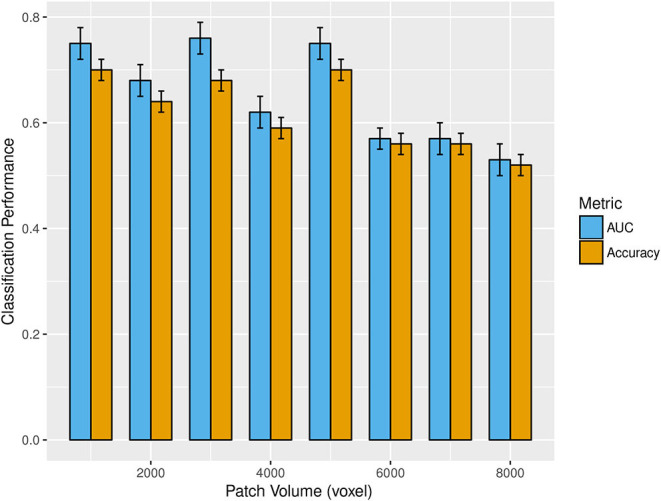
The bar plot shows the mean and standard deviations of accuracy (gold) and AUC (cyan) over 1,000 rounds of cross-validation as patch volume changes (from 1,000 to 8,000 voxels). The best classification performances were obtained at 1,000, 3,000, and 5,000 voxels.

Figure 3 shows the mean and standard deviations of accuracy and AUC over all the cross-validation rounds as a function of the patch size. The best classification performances were found at three patch volumes: 1, 000, 3, 000, and 5, 000 voxels.

Accuracy, specificity, sensitivity, and AUC with the corresponding standard deviations obtained for these three optimal scales are reported in Table 3.

**Table 3:** Accuracy, specificity, sensitivity, and AUC with the relative standard deviations obtained at the scales of 1,000, 3,000, and 5,000 voxels to which the best classification performances were reached.

**Patch volume**	**Accuracy**	**Specificity**	**Sensitivity**	**AUC**
1,000 voxels	**0.70** **±** **0.03**	**0.74** **±** **0.04**	0.66 ± 0.04	0.75 ± 0.02
3,000 voxels	0.68 ± 0.03	0.70 ± 0.04	0.67 ± 0.04	**0.76**±**0.02**
5,000 voxels	**0.70** **±** **0.03**	0.68 ± 0.04	**0.69** ± **0.04**	0.75 ± 0.02

*The highest value for the four classification metrics are reported in bold*.

**Figure 4:**
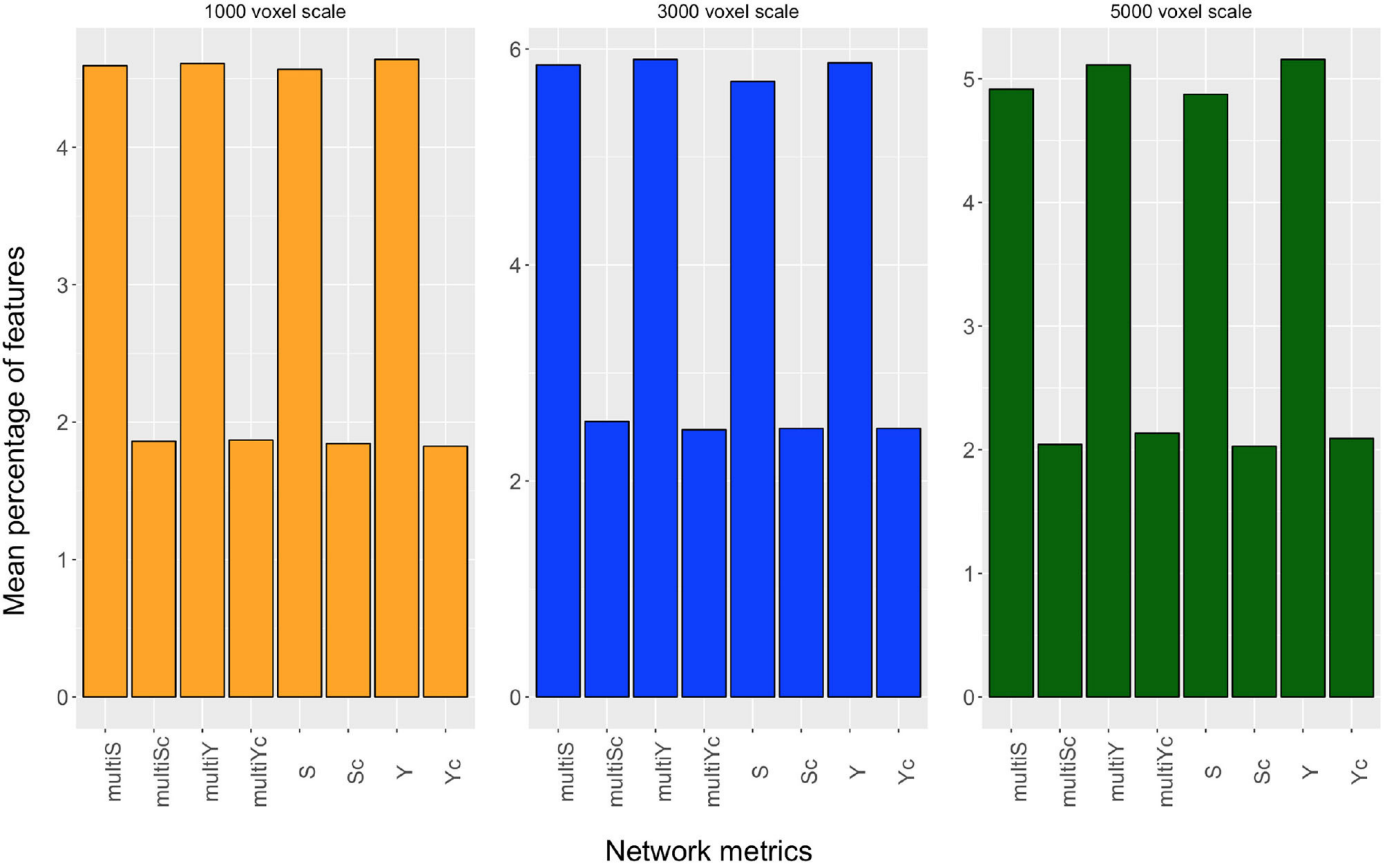
Mean percentage of features, selected over the cross-validation rounds, relative to strength (S), inverse participation ratio (Y), multi-strength (multiS), inverse-participation ratio (multiY), and their conditional means(Sc, Yc, multiSc, multiYc) are reported for the scales of 1,000 voxels (yellow barplot), 3,000 voxels (blue barplot), and 5,000 voxels (dark green barplot).

Even though we found the best accuracy for a patch size of 1,000 and 5,000 voxels (with a 95% confidence interval of [67, 73%]), and the best AUC for 3,000 voxels, the classification performances at these three scales are statistically comparable. Another important aspect is to examine which network properties are more important to discriminate the two clinical groups. In this regard, we evaluated the mean percentage of features associated with a certain network metric that are selected as important in a cross validation-round. In Figure 4, the mean percentage of features selected over the cross-validation rounds and relative to each of the eight network metrics, is reported for the three most informative scales. This experiment was performed without excluding from the classification the highly correlated features.

Even though, for each scale, all metrics extracted contribute to the discrimination of the two clinical groups, we can notice that the nodal metrics have a greater relevance compared with the conditional quantities.

### 3.2. ROI vs. Patch-Based Network Approach

**Figure 5:**
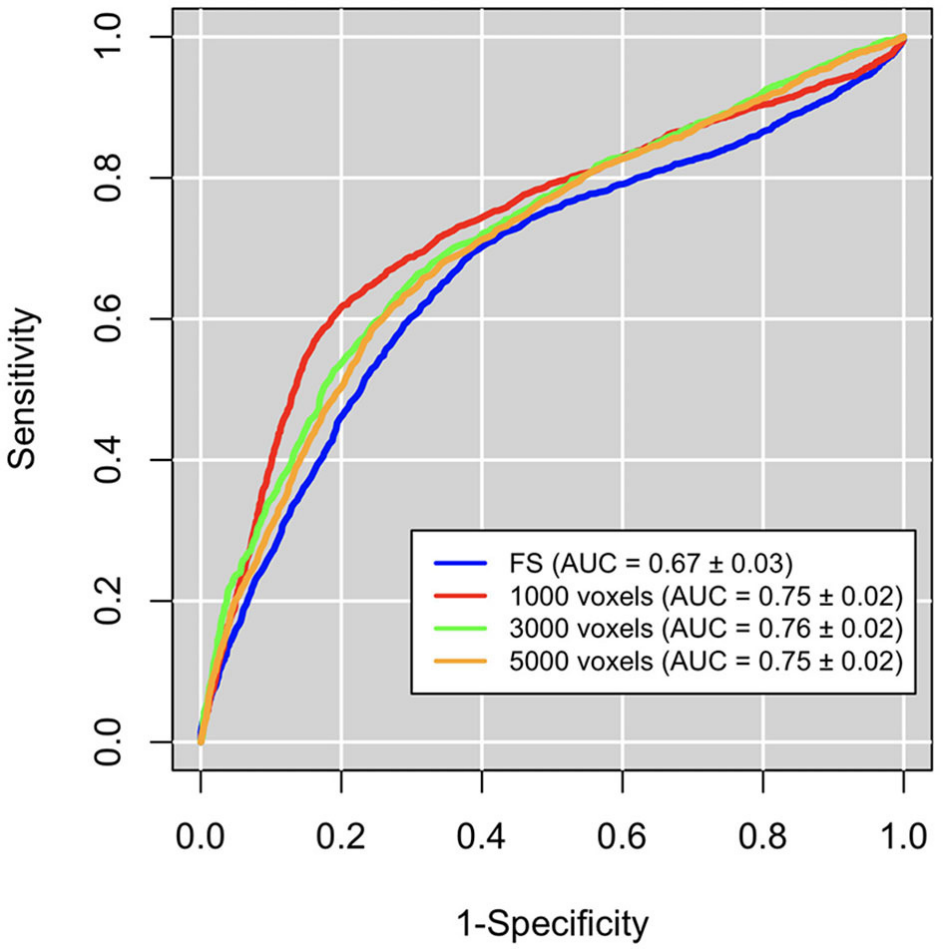
Classification performances in terms of area under the receiver operating characteristics curve (AUC). Performaces obtained with the complex network features at the the best three scales (red, orange, and green curves) are significantly greater than those obtained with FreeSurfer (FS) features (blue curves).

We also compared the patch-based network approach with a standard ROI-based approach to evaluate the efficacy of the proposed complex network methodology to predict seizure development in TBI patients. We used the publicly available brain segmentation package, FreeSurfer (FS) v.6.0 (Fischl, 2012), which automatically performs: brain extraction, intensity normalization, spatial registration, volume labeling, segmentation, and all steps necessary to compute morphological features from each image. This tool allowed us to obtain 182 features, for each MRI scan, including subcortical and cortical gray matter parcellations, white matter parcellations, total gray and white matter volumes, and intracranial volume. These FS features were then used to distinguish TBI subjects who have developed epilepsy from those who have not by adopting the same machine learning pipeline used for the complex network features. In Figure 5, receiver operating characteristics (ROC) curve and the related area under the curve (AUC) are reported for the three best scales of the complex networks and for FS.

We can notice that network approach outperform FS approach (accuracy: 0.66±0.02, sensitivity: 0.69±0.04, specificity: 0.62±0.04).

### 3.3. Anatomical Regions Related to Seizure Development

**Figure 6:**
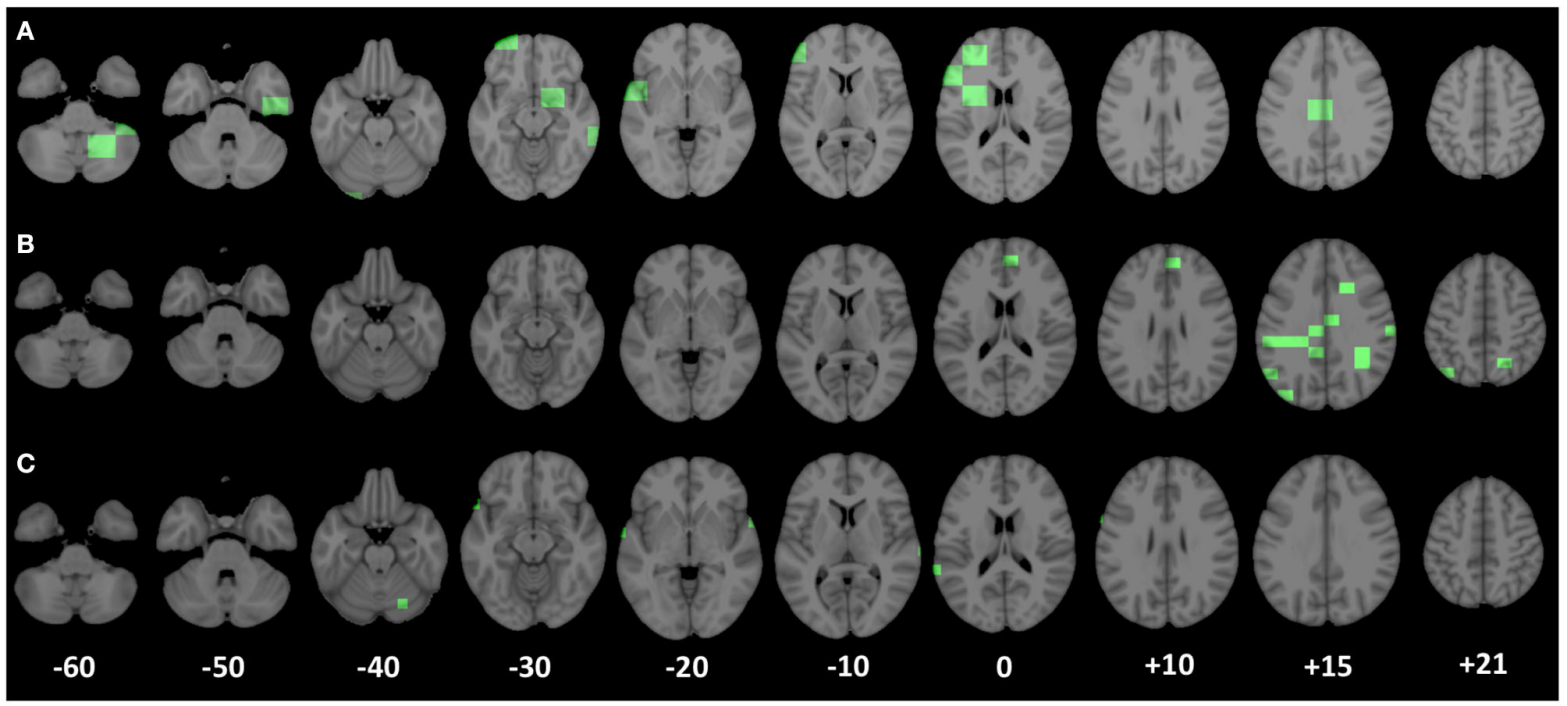
Patches corresponding to the most important nodal complex network features for the discrimination of the two clinical groups (seizure-free and seizure-affected) are underlined in green along the axial planes of the MNI 152 template. **(A–C)** Display the most significant patches relative to the scales of 5,000, 3,000, and 1,000 voxels, respectively.

For the three scales proved to be more appropriate to identify brain network alterations related to seizure development, we reported, in Figure 6, the brain areas (highlighted in green) corresponding to the most significant complex network features for the classification of seizure-free subjects and subjects with one seizure.

At the scale of 1, 000 voxels, the significant patches (*p* < 1.563*10^−5^ after the Bonferroni correction) identify anatomical regions mostly located in the right and left superior temporal gyrus lobe, but there are significant patches also in the left middle temporal gyrus, left inferior frontal and precentral gyrus, and in the right cerebellum within posterior lobe. At the scale of 3, 000, the most important brain area (*p* < 3.962*10^−5^ after the Bonferroni correction) for the two group discrimination corresponds to the cingulate gyrus in the left parietal lobe and in the right and left limbic lobe, sub-gyral in left and right frontal and parietal lobe, right and left precuneus, right postcentral gyrus, left inferior parietal lobule, angular gyrus, medial frontal gyrus, and superior occipital gyrus. Finally, the important areas (*p* < 6.361*10^−5^ after the Bonferroni correction) at the scale of 5, 000 voxels were the left and right cerebellum in the posterior lobe, right parahippocampal gyrus, right subcallosal gyrus, left inferior middle, and superior frontal gyrus, sub-gyral in the left frontal lobe, cingulate gyrus in the left limbic lobe, right and left extra-nuclear white matter, and left insula.

**Figure 7:**
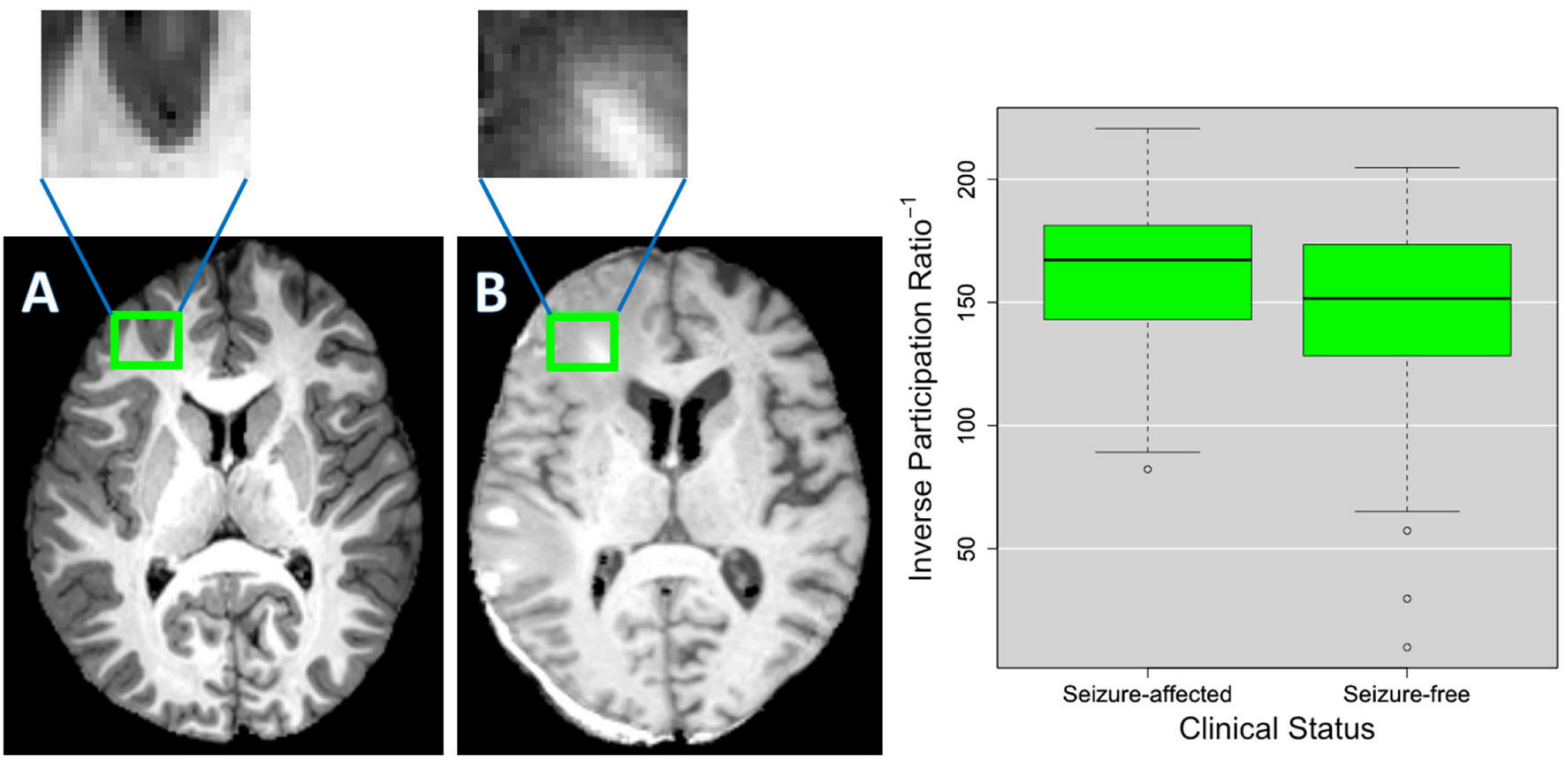
As an example, **(A,B)** show the details of a patch in two different TBI subjects. As shown in the green box plots on the right, this patch has an inverse participation ratio that is significantly different (*p* < 2.2*10^−16^) in the two clinical groups (seizure-free and seizure-affected patients). **(B)** Shows the patch pinpointing an area where voxels intensity is altered by a lesion and the surrounding edema and **(A)** shows the patch covering a brain area that does not have evident alterations.

To make more understandable the relationship between patches and complex network features, in Figure 7, the distribution of the reciprocal of the inverse participation ratio of a patch, located in the left frontal lobe, is reported for both clinical groups. In the same figure, the representation of such a patch in a seizure-affected patient who has significant abnormalities in that area and in a seizure-free patient who does not have visible anomalies in that area is shown. The inverse participation ratio relative to the patch represented in Figure 7 is an example of a network feature which is important for the discrimination of the two clinical groups. Indeed, from the box plot, we can notice that the median of the distribution for the seizure-affected subjects is significantly greater than the median of the distribution for the seizure-free subjects.

## 4. Discussion

In this work, an innovative multiplex network approach was used to find informative complex network features that can be used from machine learning systems for the identification of patients who have developed a seizure after a TBI. To the best of our knowledge, this is the first study to distinguish seizure-free subjects and seizure-affected subjects with an accuracy of 70% and an AUC of 76% by using T1-weighted MRI data. In Messori et al. (2005), PTE prediction using human MRI is based only on statistical evaluations. Classification performances obtained in this work are comparable and even higher than those found in La Rocca et al. (2019) and Garner et al. (2019) that examine functional and structural alterations related to seizure onset. All the network properties are proved to be useful to the classification, however, features relative to conditional metrics were selected less frequently in each cross-validation round. Classification performances were computed as a function of the patch volume, which was varied in an intermediate range (from 1,000 to 8,000 voxels) in order to avoid that the analysis was affected by a low sensitivity to subtle pathological changes in case of too large patches or by the 'curse of dimensionality' and misregistration in case of too small patches. The best classification performances were obtained at three different scales: 1,000, 3,000, and 5,000 voxels, proving that the study of seizure development in TBI patients requires multivariate analyses. Although some of the important anatomical regions, such as cingulate gyrus, sub-gyral, inferior central gyrus, and cerebellar tonsil in the right cerebellum are accordant for the three scales, we can notice that some morphological changes between the two clinical groups can be detected only at specific scales. This suggests that seizure development in TBI patients cannot be studied considering a unique scale, which is reasonable given the heterogeneity of the epileptogenesis process after a TBI and the fact that TBIs affect the brain in different areas and at different scales. As a consequence, analyzing multiple scales can give a more exhaustive detection of the MRI changes related to seizure development after a TBI that a unique scale is not able to provide. Therefore, our methodology can be very useful to perform a multivariate analysis that take into account multi-scale features. Conventional volumetric analyses are based on manual segmentation of the Region-Of-Interest (ROI) that is time-consuming and affected by personal bias. The modern automated algorithms that allow the determination of volumes, thickness, and shape of anatomical structures often fail because of large lesion size and extensive tissue damage in TBI patients. In this regard, we demonstrated that our approach (AUC of 76%) is more effectiveness than a ROI-based approach like FS (AUC of 67%). EpiBioS4Rx is an ongoing study that will enroll 300 patients, therefore in upcoming years, with a larger and more representative training sample, we will be able to fully exploit machine learning potentialities and obtain conclusive results about the generalization power of the model in predicting seizure development in TBI patients (Figueroa et al., 2012). Once more subjects will be enrolled and longitudinally examined, it will be also interesting to see if the proposed methodology is able to distinguish among immediate, early, and late seizures in order to take into account also the temporal aspect of the epileptogenic process. Besides, the completely automated complex network approach used in this work can be really beneficial because it allows an unsupervised identification of the important brain areas without being affected by ROI segmentation mistakes and time-consuming procedures. This methodology offers also other two main advantages: (i) it allows the identification of MRI changes which differentiate seizure-free and seizure-affected patients and which cannot be underlined using only CT MRI findings (see Table 1); (ii) it allows the identification of the brain scales at which the pathological changes related to seizure development occur. Indeed, as might be expected, given TBI variability, epileptogenesis mechanism will depend on alterations that happen at different scales (Cloots et al., 2013). It is interesting to notice that at the scale of 1,000 voxels, most of the patches are located at the periphery of the brain. This may be due to brain surface deformations or to subdural and epidural hematomas that are reported among the risk factors to develop epilepsy and are present in many subjects of this cohort as reported in Table 1 (Agrawal et al., 2006). An example of subdural hemotoma is shown in Figure 7B in the left posterior part of the brain. Most of the clinical results are in line with recent studies about seizure development. Norden and Blumenfeld (2002) states the increased likelihood of cerebellar alterations in patients with epilepsy. In Shultz et al. (2013), MRI alterations were found in hippocampus subfields of rodents with epilepsy after a lateral fluid percussion injury. Tubi et al. (2019) showed that subjects with lesions in the temporal lobe are at high risk to develop epilepsy, suggesting that morphological alterations in the temporal lobe may play a strategic role in seizure occurrence. Hippocampus, cingulate gyrus, precentral gyrus, postcentral gyrus, and middle and inferior frontal gyrus were proved to be regions related to the epileptogenesis process also in studies that apply machine learning techniques to fMRI and sMRI to characterize patients with epilepsy (Zhang et al., 2012; Garner et al., 2019; La Rocca et al., 2019). It is worthwhile to notice that the highly correlated (>0.95) features that were excluded in the classification correspond to complex network metrics related to the same patch and thus, to the same brain area. This ensures that we did not exclude any important region in the clinical validation and suggests that for some patches more complex network metrics are accordant with each other.

## 5. Conclusion

We have demonstrated that the combined use of complex networks and machine learning techniques can be useful to study seizure development in TBI patients. Multiplex networks were able to provide network features that allow us to distinguish TBI patients who have developed epilepsy from those who have not with an accuracy of 70%. In addition, a patch-based approach used to build the multiplex networks made it possible to identify, in an unsupervised way, the brain areas important for the discrimination of the two clinical groups, even though a perfect solution of optimum features is a challenging and still open matter. EpiBioS4Rx is an ongoing study that will enroll 300 patients, thus in the near future, a larger dataset will be available, and we will be able to obtain more conclusive results.

## Data Availability Statement

The data analyzed in this study is subject to the following licenses/restrictions: access to data must be requested and approved by the EpiBioS4Rx steering committee. Requests to access these datasets should be directed to epibiossteeringcommittee@loni.usc.edu.

## Ethics Statement

This work was approved by the UCLA Institutional Review Board (IRB# 16-001 576) and the local review boards at each EpiBioS4Rx Study Group institution. Written informed consent to participate in this study was provided by the participants' legal guardian/next of kin.

## Author Contributions

ML conceived and conducted the analyses. ML, RG, NA, EL, MM, PV, AT, and DD analyzed the results and reviewed the manuscript.

## Conflict of Interest

The authors declare that the research was conducted in the absence of any commercial or financial relationships that could be construed as a potential conflict of interest.

## Footnotes

^1^https://sites.google.com/g.ucla.edu/epibios4rxmobilewebsite/inclusionexclusion-criteria

^2^https://qc.loni.usc.edu

## References

[B1] Agrawal A. Timothy J. Pandit L. Manju M. (2006). Post-traumatic epilepsy: an overview. Clin. Neurol. Neurosurg. 108, 433–439. 10.1016/j.clineuro.2005.09.001 16225987

[B2] Amoroso N. La Rocca M. Bellotti R. Fanizzi A. Monaco A. Tangaro S.. (2018a). Alzheimer's disease diagnosis based on the hippocampal unified multi-atlas network (human) algorithm. Biomed. Eng. Online 17:6. 10.1186/s12938-018-0439-y 29357893 PMC5778685

[B3] Amoroso N. La Rocca M. Bruno S. Maggipinto T. Monaco A. Bellotti R.. (2018b). Multiplex networks for early diagnosis of Alzheimer's disease. Front. Aging Neurosci. 10:365. 10.3389/fnagi.2018.00365 30487745 PMC6247675

[B4] Amoroso N. La Rocca M. Monaco A. Bellotti R. Tangaro S. (2018c). Complex networks reveal early MRI markers of Parkinson's disease. Med. Image Anal. 48, 12–24. 10.1016/j.media.2018.05.004 29807313

[B5] Bharath R. D. Panda R. Raj J. Bhardwaj S. Sinha S. Chaitanya G.. (2019). Machine learning identifies “rsfMRI epilepsy networks” in temporal lobe epilepsy. Eur. Radiol. 29, 1–10. 10.1007/s00330-019-5997-2 30734849

[B6] Bianconi G. (2018). Multilayer Networks: Structure and Function. Oxford: Oxford University Press. 10.1093/oso/9780198753919.001.0001

[B7] Biau G. Scornet E. (2016). A random forest guided tour. Test 25, 197–227. 10.1007/s11749-016-0481-7

[B8] Breiman L. (1996). Bagging predictors. Mach. Learn. 24, 123–140. 10.1007/BF00058655

[B9] Cloots R. J. Van Dommelen J. Kleiven S. Geers M. (2013). Multi-scale mechanics of traumatic brain injury: predicting axonal strains from head loads. Biomech. Model. Mechanobiol. 12, 137–150. 10.1007/s10237-012-0387-6 22434184

[B10] Diaz-Arrastia R. Agostini M. A. Madden C. J. Van Ness P. C. (2009). Posttraumatic epilepsy: the endophenotypes of a human model of epileptogenesis. Epilepsia 50, 14–20. 10.1111/j.1528-1167.2008.02006.x 19187290

[B11] Figueroa R. L. Zeng-Treitler Q. Kandula S. Ngo L. H. (2012). Predicting sample size required for classification performance. BMC Med. Inform. Decis. Mak. 12:8. 10.1186/1472-6947-12-8 22336388 PMC3307431

[B12] Fischl B. (2012). Freesurfer. Neuroimage 62, 774–781. 10.1016/j.neuroimage.2012.01.021 22248573 PMC3685476

[B13] Floares A. Ferisgan M. Onita D. Ciuparu A. Calin G. Manolache F. (2017). The smallest sample size for the desired diagnosis accuracy. Int. J. Oncol. Cancer Ther. 2, 13–19.

[B14] Garner R. La Rocca M. Barisano G. Toga A. W. Duncan D. Vespa P. (2019). “A machine learning model to predict seizure susceptibility from resting-state fMRI connectivity,” in Proceedings of the Modeling and Simulation in Medicine Symposium, 14 (Tucson, AZ: Society for Computer Simulation International). 10.23919/SpringSim.2019.8732859 PMC9760283 36541915

[B15] Humphreys I. Wood R. L. Phillips C. J. Macey S. (2013). The costs of traumatic brain injury: a literature review. Clin. Econ. Outcomes Res. 5:281. 10.2147/CEOR.S44625 23836998 PMC3699059

[B16] Immonen R. Harris N. G. Wright D. Johnston L. Manninen E. Smith G.. (2018). Imaging biomarkers of epileptogenecity after traumatic brain injury-preclinical frontiers. Neurobiol. Dis. 123, 75–85. 10.1016/j.nbd.2018.10.008 30321600 PMC6818714

[B17] Jenkinson M. Beckmann C. F. Behrens T. E. Woolrich M. W. Smith S. M. (2012). FSL. Neuroimage 62, 782–790. 10.1016/j.neuroimage.2011.09.015 21979382

[B18] Kim J. A. Boyle E. J. Wu A. C. Cole A. J. Staley K. J. Zafar S.. (2018). Epileptiform activity in traumatic brain injury predicts post-traumatic epilepsy. Ann. Neurol. 83, 858–862. 10.1002/ana.25211 29537656 PMC5912971

[B19] Kriegeskorte N. Simmons W. K. Bellgowan P. S. Baker C. I. (2009). Circular analysis in systems neuroscience: the dangers of double dipping. Nat. Neurosci. 12:535. 10.1038/nn.2303 19396166 PMC2841687

[B20] Kurland D. Hong C. Aarabi B. Gerzanich V. Simard J. M. (2012). Hemorrhagic progression of a contusion after traumatic brain injury: a review. J. Neurotrauma 29, 19–31. 10.1089/neu.2011.2122 21988198 PMC3253310

[B21] La Rocca M. Amoroso N. Monaco A. Bellotti R. Tangaro S. Initiative A. D. N.. (2018). A novel approach to brain connectivity reveals early structural changes in Alzheimer's disease. Physiol. Measure. 39:074005. 10.1088/1361-6579/aacf1f 29943735

[B22] La Rocca M. Garner R. Jann K. Kim H. Vespa P. Toga A. W. (2019). “Machine learning of multimodal MRI to predict the development of epileptic seizures after traumatic brain injury,” in Medical Imaging with Deep Learning (MIDL) Abstract (London).

[B23] Lancaster J. L. Woldorff M. G. Parsons L. M. Liotti M. Freitas C. S. Rainey L.. (2000). Automated talairach atlas labels for functional brain mapping. Hum. Brain Mapp. 10, 120–131. 10.1002/1097-0193(200007)10:3<120::AID-HBM30>3.0.CO;2-8 10912591 PMC6871915

[B24] Lutkenhoff E. S. Rosenberg M. Chiang J. Zhang K. Pickard J. D. Owen A. M.. (2014). Optimized brain extraction for pathological brains (optibet). PLoS ONE 9:e115551. 10.1371/journal.pone.0115551 25514672 PMC4267825

[B25] Lutkenhoff E. S. Shrestha V. Tejeda J. R. Real C. McArthur D. L. Duncan D.. (2020). Early brain biomarkers of post-traumatic seizures: initial report of the multicentre epilepsy bioinformatics study for antiepileptogenic therapy (epibios4rx) prospective study. J. Neurol. Neurosurg. Psychiatry. 91, 1154–1157. 10.1136/jnnp-2020-322780 32848013 PMC7572686

[B26] Messori A. Polonara G. Carle F. Gesuita R. Salvolini U. (2005). Predicting posttraumatic epilepsy with MRI: prospective longitudinal morphologic study in adults. Epilepsia 46, 1472–1481. 10.1111/j.1528-1167.2005.34004.x 16146443

[B27] Mukaka M. M. (2012). A guide to appropriate use of correlation coefficient in medical research. Malawi Med. J. 24, 69–71. 10.4314/mmj.v20i1.10949 23638278 PMC3576830

[B28] Newcombe R. G. (1998). Interval estimation for the difference between independent proportions: comparison of eleven methods. Stat. Med. 17, 873–890. 10.1002/(SICI)1097-0258(19980430)17:8<873::AID-SIM779>3.0.CO;2-I 9595617

[B29] Norden A. D. Blumenfeld H. (2002). The role of subcortical structures in human epilepsy. Epilep. Behav. 3, 219–231. 10.1016/S1525-5050(02)00029-X 12662601

[B30] Shah P. Bassett D. S. Wisse L. E. Detre J. A. Stein J. M. Yushkevich P. A.. (2019). Structural and functional asymmetry of medial temporal subregions in unilateral temporal lobe epilepsy: a 7T MRI study. Hum. Brain Mapp. 40, 2390–2398. 10.1002/hbm.24530 30666753 PMC6497534

[B31] Shultz S. R. Cardamone L. Liu Y. R. Hogan R. E. Maccotta L. Wright D. K. (2013). Can structural or functional changes following traumatic brain injury in the rat predict epileptic outcome? Epilepsia 54, 1240–1250. 10.1111/epi.12223 23718645 PMC4032369

[B32] Suk H.-I. Lee S.-W. Shen D. Alzheimers Disease Neuroimaging Initiative. (2014). Hierarchical feature representation and multimodal fusion with deep learning for AD/MCI diagnosis. NeuroImage 101, 569–582. 10.1016/j.neuroimage.2014.06.077 25042445 PMC4165842

[B33] Tubi M. A. Lutkenhoff E. Blanco M. B. McArthur D. Villablanca P. Ellingson B.. (2019). Early seizures and temporal lobe trauma predict post-traumatic epilepsy: a longitudinal study. Neurobiol. Dis. 123, 115–121. 10.1016/j.nbd.2018.05.014 29859872 PMC6274611

[B34] Vabalas A. Gowen E. Poliakoff E. Casson A. J. (2019). Machine learning algorithm validation with a limited sample size. PLoS ONE 14:e0224365. 10.1371/journal.pone.0224365 31697686 PMC6837442

[B35] Verellen R. M. Cavazos J. E. (2010). Post-traumatic epilepsy: an overview. Therapy 7:527. 10.2217/thy.10.57 24761136 PMC3992621

[B36] Wilson E. B. (1927). Probable inference, the law of succession, and statistical inference. J. Am. Stat. Assoc. 22, 209–212. 10.1080/01621459.1927.10502953

[B37] Zhang J. Cheng W. Wang Z. Zhang Z. Lu W. Lu G.. (2012). Pattern classification of large-scale functional brain networks: identification of informative neuroimaging markers for epilepsy. PLoS ONE 7:e36733. 10.1371/journal.pone.0036733 22615802 PMC3355144

