# Supplementary material for: Multiplex Networks to Characterize Seizure Development in Traumatic Brain Injury Patients
Source: Front Neurosci. 2020 Nov 30;14:591662. doi: 10.3389/fnins.2020.591662 (PMC7734183; doi:10.3389/fnins.2020.591662)
Supplement: Supplementary file 1 [file Data_Sheet_1.PDF]

Supplementary Material

1 FEATURE SELECTION RELIABILITY

We carried out two tests to assess the reliability of the two feature selection methods used in this work. The first test consisted in evaluating the classification performances, for the discrimination of seizure-free and seizure-affected subjects, by running 1000 cross-validation rounds with and without the removal of null mean and variance features, and highly correlated features. Table S1 shows that without this initial feature selection the performances decrease especially for the scale of 1000 voxels (area under the receiver operating characteristics curve or AUC = 0.68 ± 0.03). As far as the scale of 3000 and 5000 voxels, we found that without the initial feature selection the AUCs are 0.71 ± 0.04 and 0.73 ± 0.02, respectively. This suggests that the initial feature selection allow the removal of redundant and confounding features especially when the feature number that the machine algorithm has to manage is larger. It is worthwhile to consider that this initial feature selection was carried out on the whole dataset but the computation of the mean and variance of the features and the computation of the highly correlated features do not involve the use of the clinical status of the subjects. Therefore, the initial feature selection is appropriately performed and does not include any bias in the classification process.

Patch volume	AUC without feature selection	AUC with feature selection
1000 voxels	0.68 ± 0.03	0.75 ± 0.02
3000 voxels	0.71 ± 0.04	0.76 ± 0.02
5000 voxels	0.73 ± 0.02	0.75 ± 0.02

Table S1. Area Under the receiver operating characteristics Curve (AUC) with the relative standard deviations obtained at the scales of 1000, 3000, and 5000 voxels with and without the removal of null mean and variance features, and highly correlated features.

The second test is about the stability of the important feature selected in each cross-validation round. We computed, over the 1000 cross-validation round, the average percentage of the important features, that are kept compared to those selected with 500 trees, as the tree number changes from 100 to 1000 in steps of 100. With this test represented in Figure, we found that after 400 trees the percentage of the important features selected remains stable. It is worthwhile to specify that, in the figure in which the percent value at 500 trees is obtained, the most important features selected at 500 trees were computed a second time.

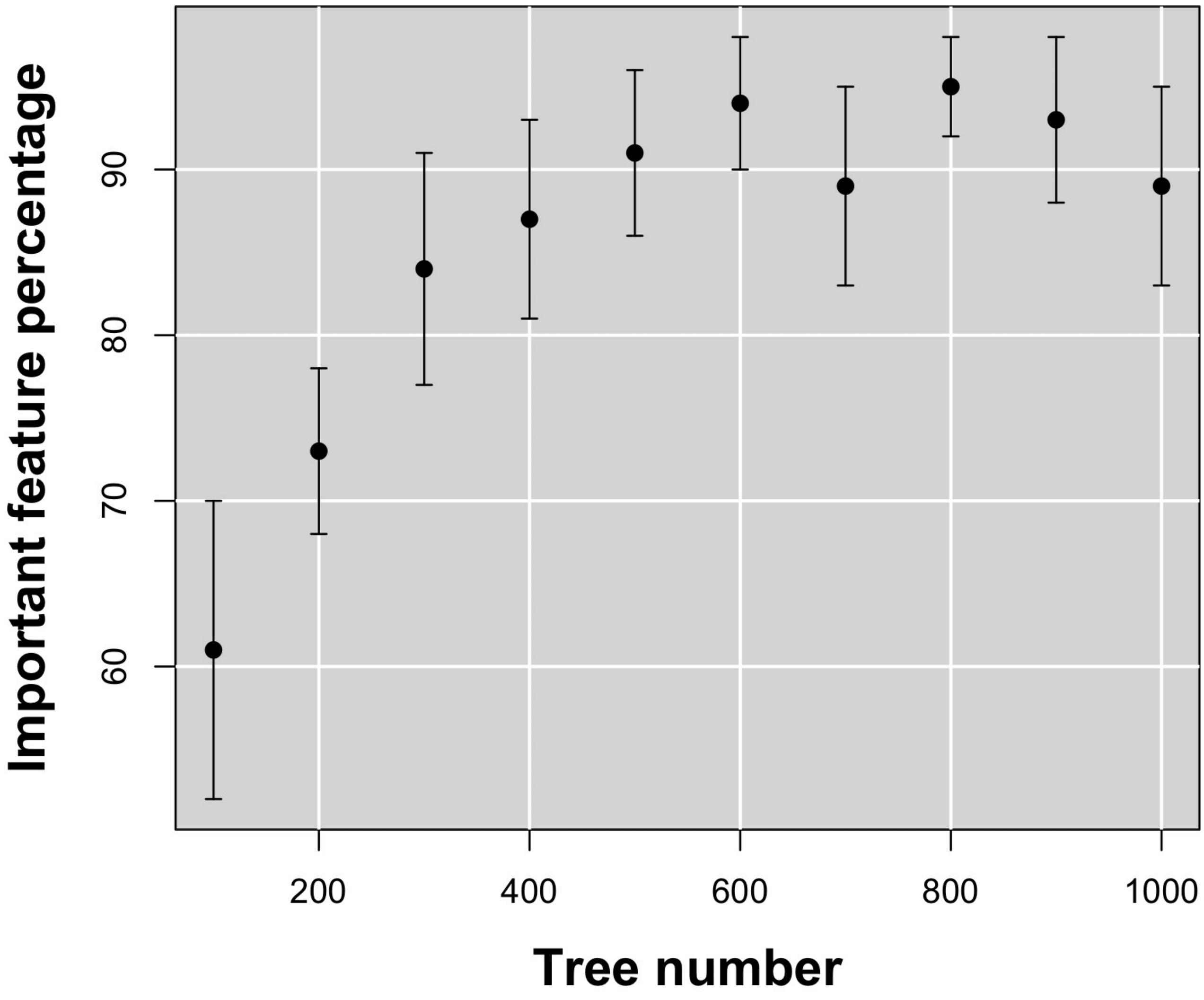

Figure S1: Average percentage of the important features that are kept over 1000 rounds of cross-validation as the tree number changes.
